# Supplementary material for: ‘Vivaldi’: an amplicon-based whole-genome sequencing method for the four seasonal human coronaviruses, 229E, NL63, OC43 and HKU1, alongside SARS-CoV-2
Source: Microb Genom. 2025 Jul 15;11(7):001451. doi: 10.1099/mgen.0.001451 (PMC12263288; doi:10.1099/mgen.0.001451)
Supplement: Supplementary Material 2. [file mgen-11-01451-s002.pdf]

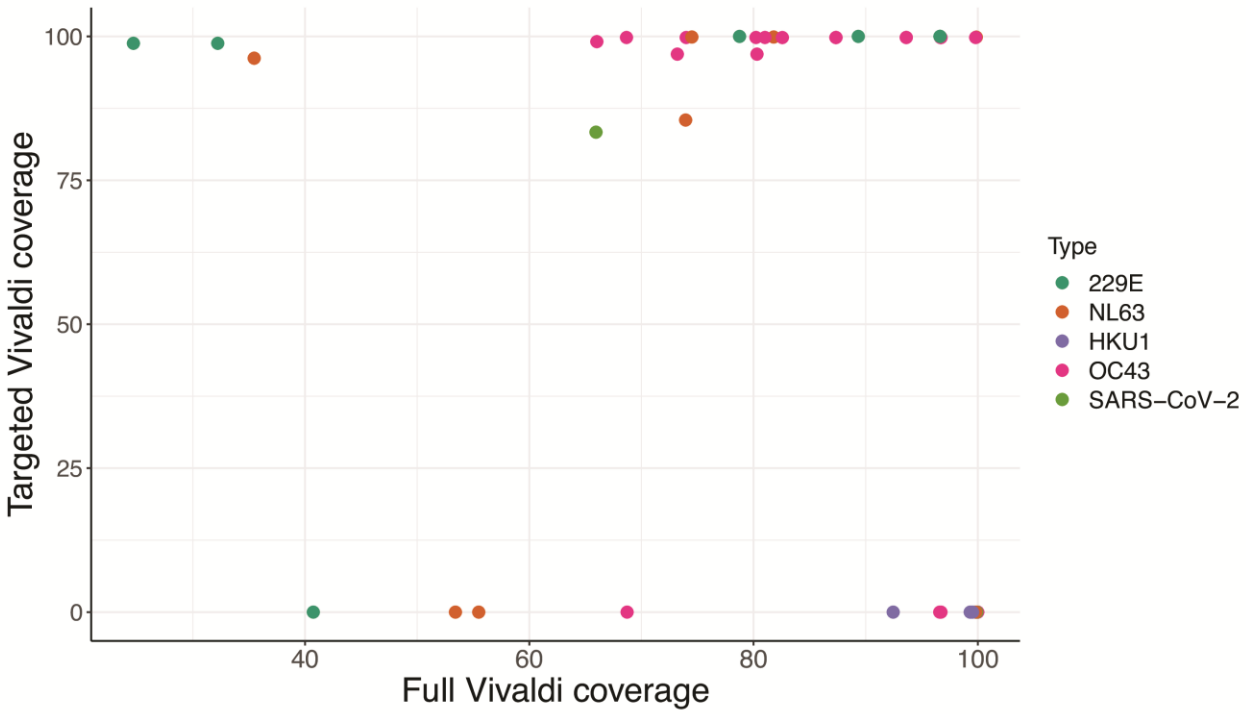

Suppl. Figure 1: *Genome coverage comparing Full Vivaldi four seasonal coronaviruses and SARS-CoV-2 combined multiplex to type-matched, targeted sequencing.* The genome coverage of 46 Seasonal Coronavirus 229E, NL63, HKU1 & OC43 and additionally SARS-CoV-2 samples when amplified by PCR multiplex containing all primer sets (Full Vivaldi, x axis), is shown compared to the genome coverage achieved with a single set of coronavirus-targeting primers (Targeted Vivaldi, y-axis). Genome coverage is expressed as a percentage of the total region targeted by the amplicon scheme. Samples with '0' coverage by the targeted method were sequenced only by the Full Vivaldi method.

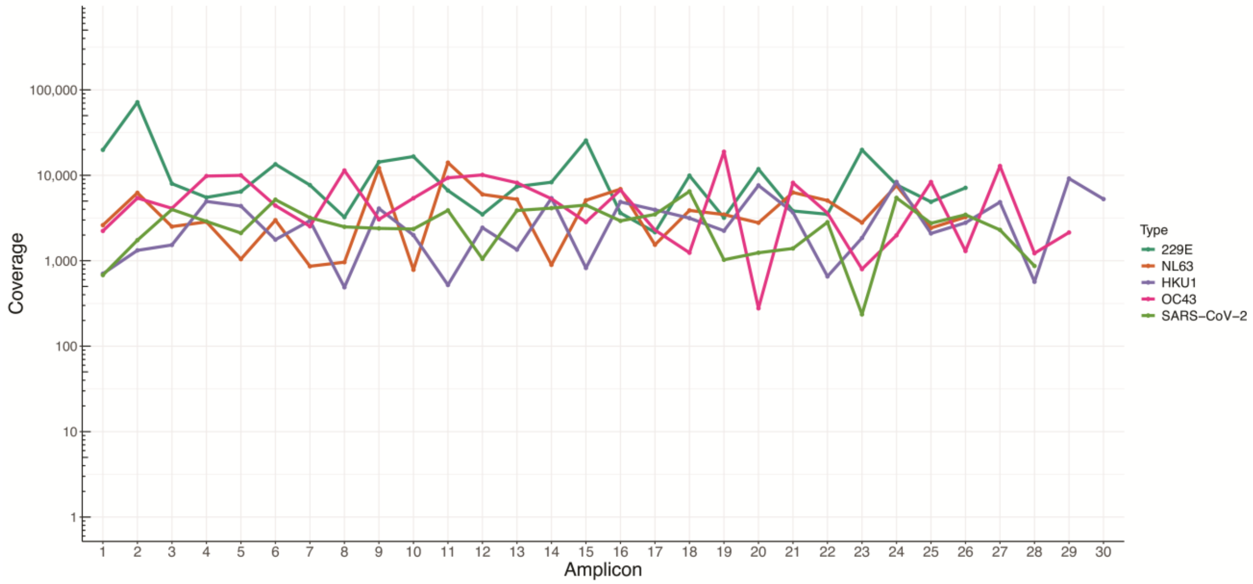

Suppl. Figure 2: *Average read depth per amplicon of optimised priming schemes.* The average number of reads (y-axis) generated across each amplicon (x-axis) derived from the final optimised primer pairs and concentrations. Total amplicon number was 26 for alphacoronaviruses 229E & NL63 and 28, 29 and 30 for betacoronaviruses SARS-CoV-2, OC43 & HKU1 respectively. Average read depth and sample size per species across all amplicons was 11373 (229E, n = 16), 4240 (NL63, n = 46), 5654 (OC43, n = 16), 3193 (HKU1, n = 21) and 2817 (SARS-CoV-2, n = 3).

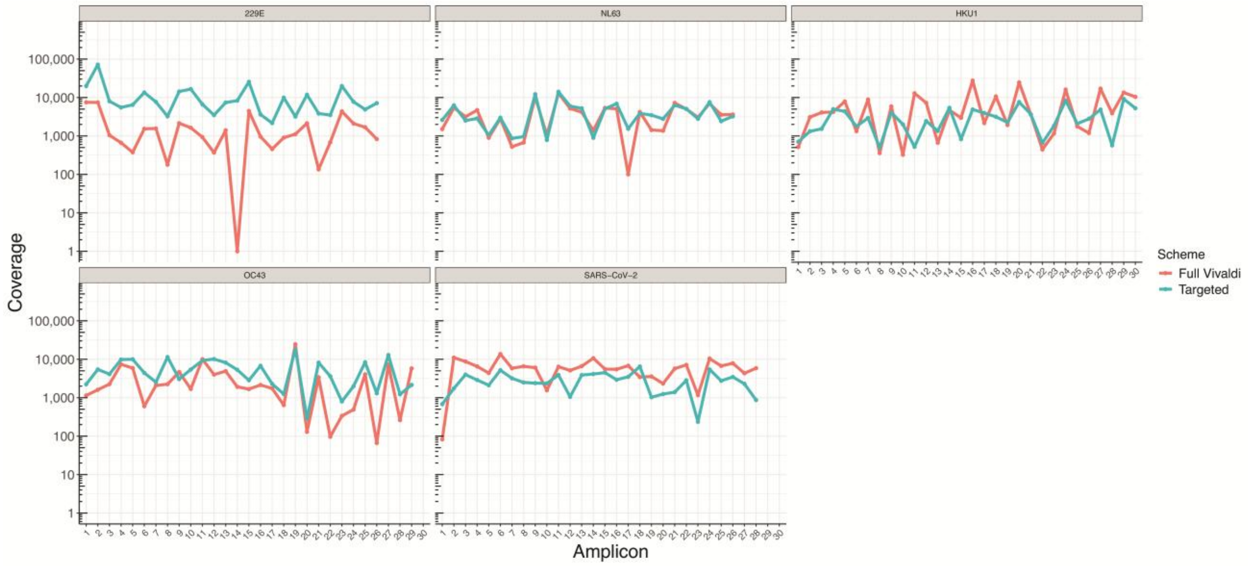

Suppl. Figure 3: *Average read depth per amplicon of Full Vivaldi compared to optimised targeted individual priming schemes.* The average number of reads (y-axis) generated across each amplicon (x-axis) from amplifications with primers targeting all five coronaviruses (the Full Vivaldi) was compared to the coverage derived from the final optimised primer pairs and concentrations specific to the presented coronaviruses (targeted): 229E, NL63, HKU1 (top, left to right), OC43 and SARS-CoV-2 (bottom, left to right). Total amplicon number was 26 for alphacoronaviruses 229E & NL63 and 28, 29 and 30 for betacoronaviruses SARS-CoV-2, OC43 & HKU1 respectively. N.B. All Full Vivaldi runs on 229E samples (n = 8) were performed on 2023 samples before a novel mismatch in the forward primer of amplicon 14 primer fsCOV_99_LEFT, which was subsequently optimised for the final scheme iteration.

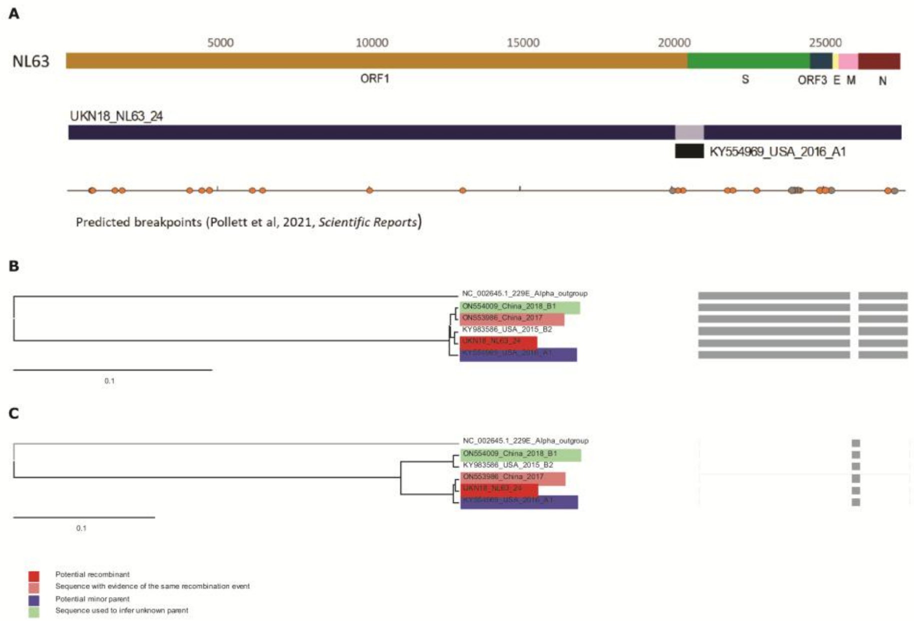

Suppl. Figure 4: *UKN18_NL63_24 recombination breakpoint analysis.* A) Genome alignments were performed on study sample UKN18_NL63_24 and reference genomes ON554009 (China 2018, Genotype B1), ON553986 (China 2017), KY983586 (USA 2015, Genotype B2, KY983586 (USA 2016, Genotype A1) and NC_002645 (229E) as an outgroup, using Clustal W with manual adjustment. Recombination was assessed using RDP4.97, with the beginning and ending breakpoints determined to be positions 19036 (p= 4.3 x10-8), and 20093 (p = 1.9 x10-14; MaxChi) respectively. Breakpoint numbers are referenced to a gap-stripped alignment. Previously reported recombination breakpoints are highlighted as described by Pollett et al, 2021 (38). UPGMA trees are shown for the major (B) and minor (C) parental sequences for UKN18_NL63_24, representing genomic regions as indicated by the grey bars to the right of each tree panel.
